# Treatment of necrotizing acute pancreatitis with peritoneal lavage and dialysis by a new simplified technique insert catheters: One retrospective study

**DOI:** 10.1097/MD.0000000000003821

**Published:** 2016-06-10

**Authors:** Qi Li, Bai Zhu, Xueyan Zhu, Chenglin Piao, Wenpeng Cui, Yangwei Wang, Jing Sun, Wenguo Chen, Lining Miao

**Affiliations:** a Department of Nephrology, The Second Hospital Of Jilin University, Changchun; b Department of Nephrology, Jilin City Central Hospital, Jilin; c Department of Gastroenterology, the First Affiliated Hospital, College of Medicine, Zhejiang University, Hangzhou, China.

**Keywords:** necrotizing acute pancreatitis, percutaneous technique, peritoneal dialysis, peritoneal lavage

## Abstract

Peritoneal lavage and dialysis is an approach to treat necrotizing acute pancreatitis as it removes dialyzable toxins and reduces severe metabolic disturbances. Successful catheter implantation is important for delivering adequate peritoneal lavage and dialysis. The aim of the present study was to describe a new modified percutaneous technique for the placement of peritoneal dialysis catheters and assess the effectiveness and safety of peritoneal lavage and dialysis used for treatment of necrotizing acute pancreatitis. We conducted a retrospective data review of 35 patients of necrotizing acute pancreatitis from January 2010 to December 2014 in Jilin City Central Hospital and The First Affiliated Hospital of ZheJiang University. In total, 18 patients underwent peritoneal lavage and dialysis after inserting catheters by our new technique (group A), whereas 17 patients underwent ultrasound-guided percutaneous catheter drainage (group B). By analyzing the patients’ data, the drainage days and mean number of hours between the debut of the symptoms and the hospital admission were lower in group A (*P* < 0.05, *P* < 0.05, respectively). The complication rate of 5.6 and 17.6%, respectively (*P* = 0.261), and a mortality rate of 16.7 and 5.9% for each group, respectively (*P* = 0.316). Likewise, hospitalization time was similar for the group A: 31 ± 25.3 days compared with 42.8 ± 29.4 days in the group B (*P* = 0.211). Peritoneal lavage and dialysis can be used in necrotizing acute pancreatitis, and our new modified percutaneous technique offers the same complication and mortality rate as ultrasound-guided drainage but with a shorter drainage days.

## 1 Introduction

Necrotizing pancreatitis is a severe form of acute pancreatitis, often causing significant morbidity and mortality. Overall mortality has been reported to be ∼8% to 39% for necrotizing pancreatitis.^[1,2]^ Efficient management is important in improving the prognosis. The recommended treatment strategy of necrotizing pancreatitis is a minimally invasive step-up approach consisting of percutaneous drainage followed, if necessary, by minimally invasive retroperitoneal necrosectomy.^[3]^

Peritoneal dialysis (PD), a successful form of renal replacement therapy, has become more common as the prevalence of end-stage renal disease (ESRD) increase. Peritoneal dialysis is an approach to treat necrotizing acute pancreatitis as it removes dialyzable toxins and reduces severe metabolic disturbances.^[4]^ Successful catheter implantation is important for peritoneal dialysis. Traditionally, there are 3 different methods for insertion of the PD catheters, including the open surgical technique, the percutaneous needle-guidewire technique, and the laparoscopic technique.^[5–7]^ We, therefore, developed a new modified technique for percutaneous PD catheters insertion. The operation is simple; no complex equipment required and does not need general anesthesia. The aim of the present study was to describe the new modified percutaneous technique for the placement of peritoneal dialysis catheters and assess the effectiveness and safety of peritoneal lavage and dialysis used for treatment of necrotizing acute pancreatitis.

## 2 Materials and methods

### 2.1 Patients

Among the 35 patients of necrotizing acute pancreatitis, we placed 18 PD catheters in 18 patients (group A) using our modified percutaneous technique at Jilin City Central Hospital from January 2010 to December 2014. After inserting catheters, we repeated peritoneal lavage until the dialysate clear, and then typically used 2 to 4 dialysis exchanges daily. In total, 17 patients (group B) underwent ultrasound-guided percutaneous drainage at The First Affiliated Hospital of ZheJiang University from January 2010 to December 2014. All patients were generally treated with supportive care including pain control, fluid resuscitation, and correction of electrolyte and metabolic abnormalities.

### 2.2 Operation method

**Figure 1:**
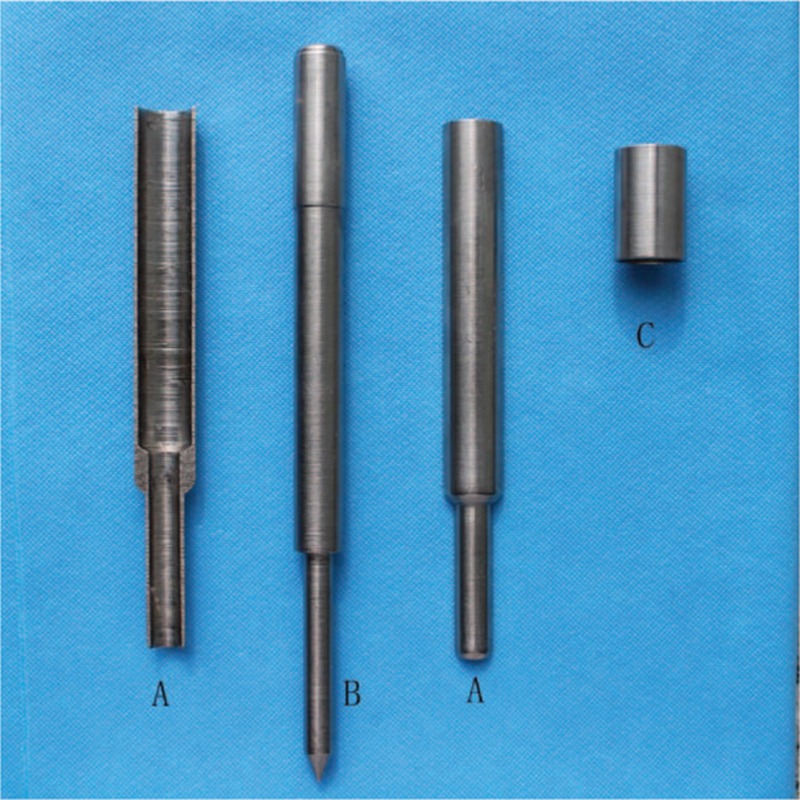
The structure of trocar: A trocar, comprising 2 independent parts; B core-needle; C hoop.

We will mainly introduce the operation procedure of our technique. The modified percutaneous technique was used based on a new trocar (Patent No. 02273526.7), which consists of 1 core-needle, a 2-part trocar, and 1 hoop (Fig. 1). The device is reusable and, therefore, cost-effective. A 2-cm paramedian longitudinal incision was made 1 to 2 cm below the umbilicus, followed by blunt dissection of the subcutaneous tissue until the fascia anterior to the rectus muscle was reached. A small incision was made, followed by a purse-string suture, temporarily not tightened. The peritoneum was then punctured using the trocar. A guidewire (supple, 60 cm long and 2 mm thick) was inserted into the peritoneal cavity through the trocar after removing the core needle. The PD catheter was then inserted over the guidewire, surrounding the hollow drivepipe and directed toward the right or left pelvic gutter. The guidewire was then removed. Finally, the PD catheter was gradually advanced toward its goal, whereas the 2-part drivepipe was removed after disentwined the hoop. The inner cuff of the PD catheter was secured with a purse-string suture on the fascia anterior to the rectus muscle. The original incision was then closed, and the PD catheter was flushed several times with a total of 2 L peritoneal dialysis solution to confirm catheter unobstructed and to check for intra-abdominal bleeding. All operations were performed by the nephrologists in the day surgery room with the patient under local anesthesia.

### 2.3 Statistical analysis

The descriptive data were expressed as absolute numbers and percentages for categorical variables and mean ± SD for quantitative variables. The chi-square test was used to compare the categorical variables; Fisher's exact test was used if the frequency <5. The Mann–Whitney *U* test was done to compare the continuous variables. We considered *P* < 0.05 as statistically significant. Statistical analysis was performed with SPSS version 19.0 for Windows.

### 2.4 Ethics statement

This retrospectively study was approved by the Ethics Committee of the First Affiliated Hospital of ZheJiang University and Jilin City Central Hospital; and written informed consent was obtained from all of the enrollees.

## 3 Results

A total of 35 patients with necrotizing acute pancreatitis were analyzed, 18 of which were treated with peritoneal lavage and dialysis by our simplified technique insert PD catheters and 17 underwent ultrasound-guided percutaneous drainage. There were 24 (68.6%) men and 11 (31.4%) women with a mean ± SD age of 48.8 ± 14.2 years. Gallstones were the most common etiology of pancreatitis. Table 1 summarizes the baseline clinical characteristics of patients according to the treatment group.

**Table 1:**
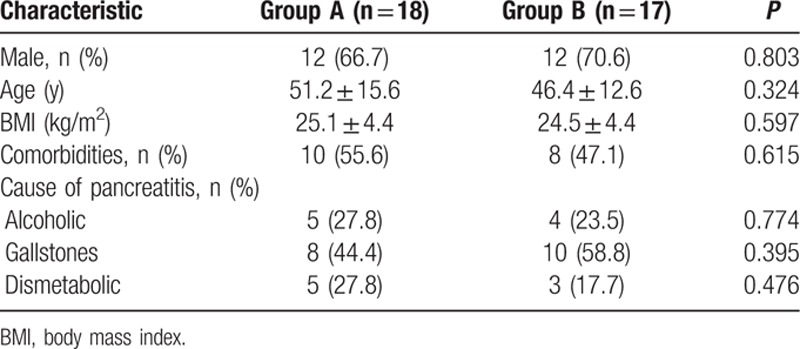
Baseline clinical characteristics of patients according to the treatment group.

The drainage days and mean number of hours between the debut of the symptoms and the hospital admission were lower in group A (*P* < 0.05, *P* < 0.05, respectively). The complication rate of 5.6 and 17.6%, respectively (*P* = 0.261), and a mortality rate of 16.7 and 5.9% for each group, respectively (*P* = 0.316). Likewise, the hospitalization time was similar for the group A: 31 ± 25.3 days compared with 42.8 ± 29.4 days in the group B (*P* = 0.211). The outcomes of 2 groups are shown in Table 2. One (5.6%) patient bled in group A; however, 1 (5.9%) patient infected and 2 (11.8%) patients existed tube plugging in group B. Complications in both groups are shown in Table 3.

**Table 2:**
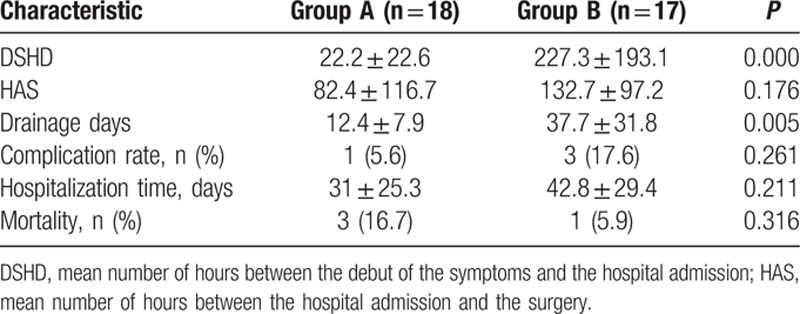
Outcomes of 2 treatments.

**Table 3:**
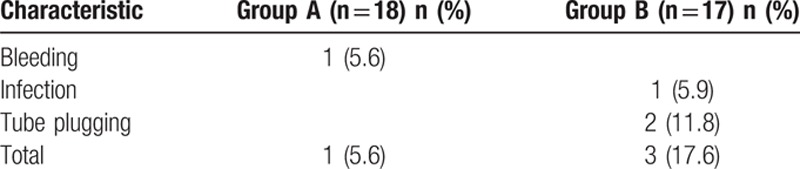
Complications according to the treatment group.

## 4 Discussion

Peritoneal lavage and dialysis is performed after abdominal surgery in necrotizing acute pancreatitis.

After inserting catheters, we repeated peritoneal lavage until the dialysate clear, and then typically used 2 to 4 dialysis exchanges daily. According to our data, there are no obvious differences in HAS, hospitalization time, complication, and mortality rate between 2 groups in the treatment of necrotizing acute pancreatitis. The complication rate of 5.6 and 17.6%, respectively (*P* = 0.261), and a mortality rate of 16.7 and 5.9% for each group, respectively (*P* = 0.316). The reasons why high complication rate with low mortality in group B are (1) 2 cases occurred drainage-tube plugging and did not have a significant influence on treatment after reposition the tube; (2) 4 patients left the hospital without fully recovered, which may have an effect on the final statistics; (3) the data analysis of sample number was small—this also has an effect on the statistics. DSHD and drainage days were significantly higher in group B. DSHD was higher in group B, probably because The First Affiliated Hospital of ZheJiang University is superior and part of patients treated with referral. Compared with group B, hospitalization time was shorter in group A, but without statistical significance. It should be considered that 4 patients of ultrasound-guided percutaneous drainage discharged the drainage tube outside the hospital. Also, the data analysis of cases was small, large sample research is needed to judge the finding. If increase the number of samples, it may turn out more statistically reliable results. The drainage days was shorter in group A, probably related to the following reasons: (1) the pipe of our simplified technique derived from Baxter used for peritoneal dialysis, the pipe diameter is obvious larger than that of the ultrasound-guided drainage group, and could wash abdominal cavity repeatedly and drainage thoroughly; (2) Regular peritoneal dialysis daily could help to remove inflammatory mediators and some toxins, further helpful to the illness recovery. In group A, 1 (5.6%) patient occurred hemorrhage in the process of operation due to vessel injury, and there was no bleeding postoperative. In the group of ultrasound-guided percutaneous drainage, 1 (5.9%) patient appeared tube infection, 2 (11.8%) cases of drainage tube obstruction, and did not have a significant influence on treatment after reposition the tube.

There are 4 main types of pancreatic collections, which include acute fluid collections (AFCs), acute necrotic collections (ANCs), pseudocysts, and walled-off necrosis (WON).^[8,9]^ AFCs develop <4 weeks after an episode of interstitial pancreatitis. They are found in the pancreatic parenchyma or peripancreatic tissue. When a fluid collection develops in the context of pancreatic necrosis, it is known as an ANC.^[10,11]^ Less invasive techniques, such as percutaneous drainage, mini- and minimally invasive necrosectomies (step-up approach) have increasingly been used with seemingly reduced mortality and morbidity.^[3,12]^ One randomized trial confirmed that a step-up approach of percutaneous catheter drainage with subsequent minimally invasive surgical necrosectomy (by endoscopic, sinus tract endoscopy, video-assisted retroperitoneal debridement) is superior to primary open necrosectomy.^[3]^ The step-up approach reduced multiple organ failure and long-term complications such as diabetes and the need for pancreatic enzymes. In addition, minimally invasive techniques have a lower complication rate than the open surgical necrosectomy rate.^[13–16]^

Insert PD catheters and keeping the tube free of obstructions is crucial to drainage. Various techniques for insertion of a peritoneal dialysis catheter have been described: the open surgical technique,^[5–7]^ the Seldinger technique, and the laparoscopic technique. There is no evidence to support a method of insertion over another. Our modified percutaneous implantation of peritoneal dialysis catheters is a safe technique and has the following advantages: simple, no complex equipment required and does not need general anesthesia. This procedure, which could easily be performed under local anesthesia, increases the commitment between the PD team and the patient, and reduces the cost and inconvenience to the patient.

Although the present study is not a randomized controlled study and the selection of patients may have been biased in many aspects, also there are still unanswered questions regarding timing and indications for intervention, our study indicated that peritoneal lavage and dialysis can be used in necrotizing acute pancreatitis and our modified percutaneous implantation of peritoneal dialysis catheters is simple and safety.
